# Human capital, technological progress and industrial restructuring

**DOI:** 10.1371/journal.pone.0325978

**Published:** 2025-06-30

**Authors:** Kui Zhao, Luyao Zhang

**Affiliations:** 1 Vocational Technology, Anhui Business College, Wuhu, China; 2 School of Finance, Tianjin University of Finance and Economics, Tianjin, China; Shanghai University of Finance and Economics, CHINA

## Abstract

Human capital and technological progress drive industrial restructuring toward rationalization and advancement, clarifying their theoretical interaction and examining the influence mechanism based on China’s three major industries data from 2008 to 2022, employing the PVAR and Hansen threshold models. The study shows that: (1) technological progress and industrial restructuring are mutually causal, while technological progress and human capital, and human capital and industrial restructuring are not mutually causal. (2) Within the research cycle, human capital has a ‘fast then slow then smooth’ influence on industrial structural adjustment, while technological progress has a ‘V’ influence on industrial structural adjustment, and technological progress has a ‘U’ influence on human capital. (3) Human capital and technological progress have played a facilitating role in industrial structural adjustment. Technological progress serves as the threshold, resulting in a double threshold effect of human capital on industrial structure rationalization, a single threshold effect is observed in its advancement. The policy implications are as follows: first, accelerate the mechanism of industrial structure upgrading, and drive the quality improvement and structure optimization of industrial human capital; second, give full play to the iterative role of technological innovation, and build a ‘technology-capital’ complementary mechanism; third, ‘according to the time - according to the place - according to the production’, Integration of human capital, technological progress and industrial restructuring synergistic development paths.

## 1. Introduction

The Global Human Capital Trends Report 2024 emphasizes the necessity of adopting innovative approaches to enhance human value creation, leveraging artificial intelligence to boost talent capabilities, and facilitating the continuous evolution of human capital through technological advancements. Simultaneously, the rapid advancement of artificial intelligence is significantly accelerating the transformation and upgrading of industrial structures, as evidenced by a multitude of applications across various sectors. and explore the evolution of the industrial chain across the globe, where the deep integration of human capital with technological progress emerges as a fresh impetus for future industrial revolutions.

As an important part of the global emerging economy, China boasts notable advantages in both the stock and increment of human capital. The new scientific and technological revolution has led to significant advancements in technological innovation, while industrial structural adjustment has reached a more profound stage, placing higher demands on the quality of human capital and technological innovation capacity, China is accelerating the creation of new quality productivity to drive the deep transformation of industrial the structure effectively, and fully leverage the combined functions and synergistic effects of various production factors. At the same time, the downward pressure on the global economy is increasing, and the external environment for China’s industrial restructuring is not optimistic. The objective facts of one positive and one negative point to a new research proposition: as a post-haircut country, how to promote the high-quality development of the economy through industrial structural adjustment, and realise the leap from ‘bending the road to overtake’ to ‘changing the road to overtake’, and following this line of thought, this paper takes China’s three major industries as the research object, and takes the human resources as the research object. Taking China’s three major industries as the research object, this paper incorporates human capital, technological progress and industrial restructuring into a unified research framework, focusing on three issues: first, elaborating the interaction mechanism of human capital, technological progress and industrial restructuring from the theoretical perspective, and clarifying the internal logic of the synergistic development of the three; second, constructing corresponding research models from the empirical level, analysing the influence mechanism of industrial restructuring, and clarifying the quantitative and quantitative results of human capital, technological progress and industrial restructuring. Secondly, we construct corresponding research models from the empirical level, analyse the influence mechanism of industrial structure adjustment, and clarify the quantitative relationship between human capital, technological progress and industrial structure adjustment; thirdly, we put forward feasible policy suggestions for the synergistic development of human capital, technological progress and industrial structure adjustment from the practical level.

For high-quality economic development, the optimization of industrial structure is fundamental, as evidenced by empirical studies showing its significant positive impact on economic growth. Industrial technology progress acts as a catalyst, while the enhancement and optimization of industrial human capital quality provide a foundational guarantee. The marginal contributions of this study are: firstly, to examine the mechanism of industrial structure adjustment by human capital, technology and other factors, to explore the dynamic evolution characteristics of factor allocation driving industrial structure towards rationalisation and advancement, and to expand the content of research on the theory of production factor allocation. Secondly, using the sample data of Chinese industries as a carrier, we analyze the influence mechanism of industrial structure adjustment, investigate the temporal relationship among human capital, technological progress, and industrial structure adjustment, and unveil the fundamental law governing the evolution of industrial structure, and the relevant findings will provide empirical evidence supporting the upgrading of industrial structures in various emerging economies globally.

## 2. Literature review and theoretical analysis

### 2.1. Policy background

The Third Plenary Session of the 20th CPC Central Committee proposed to improve the institutional mechanism for promoting high-quality economic development, and Further recognized as foundational to the development of new-quality productive forces are revolutionary technological advancements, innovative approaches to production factor allocation, profound industrial transformation and upgrading, and the synergistic integration of human capital. in accordance with local conditions, and it is necessary to profound.We must fully grasp that industrial structural adjustment serves as a pivotal breakthrough in advancing high-quality economic development, with human capital and technological progress constituting the linchpins of this transformation. Indeed, human capital and technological advancements are the cornerstone elements driving the process of industrial restructuring. According to the data of China Human Capital Report (2023), the total human capital of China continues to grow, and the total value of human capital will reach 3378.3 trillion yuan in 2021, and a notable disparity exists in human capital quality among the three major regions. Specifically, the proportions of human capital quality in the central and western regions are 91.52% and 85.25% of that in the eastern region, respectively. At the same time, under the influence of the global industrial chain and supply chain resilience, China’s industrial structural adjustment is facing the doubt.Under the squeeze of ‘high-end reflux’ and ‘middle- and low-end diversion’, the potential risk of decreased industrial technological innovation looms large. Furthermore, the human capital structure across the three major industries is markedly differentiated. These observations, coupled with the aforementioned policy signals and research reports, suggest that there are significant disparities in the human capital quality among the three major regions. The above policy signals and research reports indicate that research on the relationship between human capital, Technological advancements and industrial restructuring hold immense importance in overcoming obstacles during the optimization and upgrading of industrial structures, efficiently unleashing the potential of human capital, technology, and other production factors, thereby achieving high-quality economic development.

### 2.2. Literature review and theoretical analysis

#### 2.2.1. Human capital and industrial restructuring.

The theorem of Matchday-Clark first explored the relationship between industrial structure and labour force employment, pointing out that economic development, per capita national income levels, and the basic law of inter-industry income differences drive the workforce to shift from the primary industry to the secondary and tertiary industries [1]. Extending the perspective of industrial structure change, Kuznets revealed that the proportion of labour force in primary and secondary industries declines, the proportion of labour force in tertiary industry rises and the proportion of national income accounted for by different industries, and the change of the proportion of labour force employment in industry is an important basis for measuring the adjustment of industrial structure [2].

Empirical data from different countries have empirically examined the relationship between human capital and industrial structural adjustment, and some scholars, through the comparative study of developed and developing countries, believe that industrial structural adjustment and labour force transfer in developed countries are basically synchronous, but the speed of labour force employment structure conversion in developing countries is lagging behind the industrial structural adjustment, which results in the imbalance of industrial structure and human capital allocation [3],mainly manifesting that For human capital input and use of the situation restricts the speed and direction of industrial structural adjustment [4], similarly, industrial structural adjustment will affect the human capital through the ‘diffusion effect’, ‘compensation effect’, ‘destructive effect Similarly, industrial structural adjustment will affect the change of human capital structure through ‘diffusion effect’,‘compensation effect’,‘destruction effect’ and other ways [5].

There are three basic paths through which industrial structural adjustment affects human capital structure: first, industrial structural adjustment results in the elimination of some old industries, gives rise to new industries, and drives the development of related industries, thus expanding total social demand, increasing employment opportunities for the labour force, and stimulating the demand for human capital. Secondly, industrial restructuring promotes the rationalisation and advancement of industries through technological progress. On the one hand, the new technology substitutes for the original labour force, which makes some labour-intensive industries transform to capital- and technology-intensive industries, and reduces the demand for human capital; on the other hand, the new technology improves the industry’s total factor productivity, which guides the individuals, enterprises, and industries to make additional investment in human capital, and adapts to the future demand for industrial talents, which promotes the improvement of human capital quality and enhances human capital demand. Promote the quality of human capital and enhance the efficiency of human capital market flow. Thirdly, by the common effect of government regulation and market mechanism, industrial structural adjustment drives urban and rural, regional and industrial human capital allocation differentiation, research shows that the excessive concentration of human capital, spatial and temporal mismatch has slowed down the speed of industrial structural transformation and upgrading efficiency [6–8],especially in countries and regions with post-haircut economies, industrial structural adjustment and human capital accumulation follow different paths, and industrial upgrading should drive the progress of human capital, thus forming a virtuous circle of industrial upgrading. Industrial upgrading should be used to drive the progress of human capital, and then form a benign matching mechanism [9].

#### 2.2.2. Human capital and technological progress.

Classical growth theories and modern economic theories alike have fully affirmed the significant contribution of human capital and technological progress to economic growth. Moreover, the concept of economic growth has evolved from ‘balanced’ to ‘optimal’, and ultimately to ‘high-quality’. Throughout this transformation, the interplay between human capital and technological progress has undergone profound changes.

First, technological progress has an iterative promotion effect on human capital [10]. Artificial intelligence, as a symbol, stimulates the differentiation of industrial human capital structure through its conduction and spillover effects. Emerging industries create strong demand for high-skilled labor, while at the same time squeezing out part of the low-skilled human capital, and two-way stimulation promotes the upgrading of the human capital structure and the formation of the intrinsic circular mechanism of human capital mechanism [11,12]. In addition, the application of artificial intelligence technology will enhance the human capital ‘dry middle school’ phenomenon, altering the premium levels across various skill levels in the labour market, with the premium space shrinking in the low-level market and expanding in the high-level market, in relative terms, the positive externality effect of high-level human capital on technological progress is greater than that of low-level human capital [13].

Second, human capital is the cornerstone of technological innovation and the vehicle for its advancement. Schultz posits that education is the foundational element in the creation of human capital, and that global investment in education is essential to elevate the skill level of the workforce, thereby ensuring a plentiful supply of high-quality human capital to fuel technological innovation and economic prosperity.At the same time, urbanisation, which is characterised by population migration, also plays a role in promoting the quality of human capital, and the movement of population from rural to urban areas generates the spatial agglomeration effect of human capital and the spillover effect of knowledge and technology. since the 2lst century, China’s university expansion policy and urbanisation have created favourable conditions for the leapfrog progress of human capital from quantitative to qualitative change, and the increase in the total amount of human capital has brought into full play the advantages of the demographic dividend, and the quality of human capital has been improved. The improvement of human capital quality has promoted the in-depth integration of talent and technology, and effectively promoted the kinetic energy of economic growth [14,15].

Third, empirical studies have shown that there is an interactive mechanism between industrial technological progress and human capital [16], initially, technological advancements propel the overall labor productivity within the industry, while the mechanism for technological upgrading accelerates the renewal of industrial human capital, which is conducive to the optimization of the structure of human capital at different levels. Secondly, by strengthening the investment in basic human capital education, rationally allocate industrial human capital resources, optimize the structure of industrial talent supply, and enhance the efficiency of the talent market flow mechanism, actively adapting to the direction of industrial digitalisation and intelligent development, and forming the ‘capital-technology’ endogenous complementary mechanism, so as to realise the full employment in the labour market, and to provide a better environment for the industry. Full employment in the labour market, providing an important opportunity for the high-quality development of industrial human capital [17].

#### 2.2.3. Human capital, technological progress and industrial restructuring.

Endogenous growth theory views technological progress as crucial for sustained economic growth, regarding it as the internal force driving industrial structural adjustment, which in turn prompts shifts in the industrial human capital structure. Consequently, an increasing number of scholars have deeply recognized the importance of examining the interplay between industrial structure, human capital, and economic growth from a multi-dimensional perspective. Based on the viewpoint of coupling theory, it is believed that the coupling of industrial structure and human capital structure has a high degree of coordination, and the effect of human capital and positive externalities can enhance the efficiency of technological progress, and the match between human capital and technological capital has a significant positive impact on industrial restructuring [18,19]. Based on the viewpoint of the mechanism of action, it is believed that there are threshold effects, mediation effects [20]and moderating effects [21] of human capital, technological progress and industrial structure adjustment. First, the mechanism of the role of technological progress on industrial structure upgrading is correlated with the degree of human capital advanced, and the deep integration of artificial intelligence and high-skilled human capital can significantly promote industrial structure adjustment [22]. Secondly, human capital acts on industrial structure adjustment through the conduction path of technological progress, strengthens education investment and technological research and development, optimises the human capital structure with AI application, improves human capital efficiency, and then promotes the industrial structure towards rationalisation and advanced [23,24].There are also based on the heterogeneity perspective to analyse the specific path of the two influences on industrial structure change, pointing out that the synergistic effect of human capital and technological innovation can help accelerate industrial structure adjustment according to local conditions [25].

Based on theoretical elaboration and normative analysis, this paper summarises the relationship between human capital, technological progress and industrial structural adjustment as follows: firstly, human capital and technological progress drive industrial structural adjustment, with quality enhancement and structural optimisation of human capital ensuring the rationalization and advancement of industrial structure, potentially subject to a certain threshold.he impact of technological progress on industrial structural adjustment. Secondly, industrial structural adjustment triggers simultaneous changes in human capital, enhances the flow and allocation efficiency of industrial human capital, and then accelerates the structural differentiation of human capital, and Technological advancements, such as the development of artificial intelligence, have been instrumental in fostering the birth of new industries and reshaping industry ecosystems, thereby accelerating the pace of industrial structural adjustment. Thirdly, technological progress is heavily dependent on human capital, and the effective matching between technological progress and human capital is crucial.Progress and human capital may exhibit a more pronounced impact mechanism on industrial structural adjustment.

## 3. Research design

### 3.1. Modelling

#### 3.1.1. Panel vector auto-regressive modelling (PVAR).

It is suitable for examining the dynamic relationship of panel data, endogenising the variables by building a matrix vector and unit root test to overcome individual and time effects, with the basic expression:


$$Y_{t}=C+A_{1}Y_{t-1}+A_{2}Y_{t-2}+...+A_{p}Y_{t-p}+\mu_{t}$$ (1)

Y_t_ denotes the n-dimensional vector of endogenous variables, consisting of the natural logarithms of the study variables forming the vector set Y_t_=(lnIR, lnTP, lnHR), C denotes the n-dimensional vector of constant terms (n = 3), A_1_,...,A_p_ denotes the n*n dimensional coefficient matrix, p denotes the maximum lag order, and µ_t_ denotes the random error term.

#### 3.1.2. Hansen threshold panel model (PTR).

The model addresses individual fixed effects by removing the time-mean variance, and employs OLS (Least Squares) estimation to detect the presence of a single threshold effect, determined for a specified threshold value. In cases of double or multiple thresholds, prioritize fixing one threshold, subsequently apply the Bootstrap method to establish the next, and revert to the single threshold testing procedures for significance assessment. The basic expression of the single threshold regression model is:


$$y_{it}=\left\{ \begin{array}{c} \alpha_{i}+\beta_{1}x_{it}+e_{it}\left(q_{it}\leq \gamma \right)\\ \alpha_{i}+\beta_{2}x_{it}+e_{it}(q_{it}>\gamma ) \end{array}\right.$$ (2)

y_it_ denotes the explanatory variable (IR), x_it_ denotes the explanatory variable (TP), q_it_ denotes the threshold variable (HR),α_i_ denotes the constant term, β_1_ and β_2_ denote the estimated parameters, e_it_ denotes the residual term,γdenotes the threshold value. A basic function F() is constructed based on the threshold value, if qit ≤ γ,F()=1, if q_it_ ≥ γ,F()=0. The corresponding control variables (X) are added to the actual model,and equation (2) is transformed into:


$$y_{it}=\alpha_{i}+\beta_{1}x_{it}F(q_{it}\leq \gamma )+\beta_{2}x_{it}F(q_{it}>\gamma )+\theta X+e_{it}$$ (3)

By analogy, the basic expression for the double threshold regression model is:


$$y_{it}=\alpha_{i}+\beta_{1}x_{it}F(q_{it}\leq \gamma_{1})+\beta_{2}x_{it}F(\gamma_{1}<q_{it}\leq \gamma_{2})+\beta_{3}x_{it}F(q_{it}>\gamma_{2})+\theta X+e_{it}$$ (4)

### 3.2. Variable measurement and description

#### 3.2.1. Explained variable: Industrial restructuring (IR).

The measurement of industrial structure adjustment is quantified through the product of the industrial structure rationalisation index (IR_m_) and the industrial structure advanced index (IR_q_) [26]. This approach is grounded in the understanding that the evolution of industrial structures is a critical determinant of economic growth, as evidenced by empirical studies that highlight the interplay between structural changes and economic development.


$$IR_{m}=\frac{\left[\sum_{i=1}^{n}\left|e(i,t+1)-e(i,t)\right|\right]-\left|e(t+1)-e(t)\right|}{e(t)}$$ (5)

$$IR_{q}=\sum \left(S_{ijt}*F_{ijt}\right)/100$$ (6)

e (i,t), e (i,t + 1)denotes the number of people employed in industry in region i in period t and t + 1, e (t), e (t + 1)denotes the number of people employed in all industries in region i in period t and t + 1, S_ijt_ denotes the share of value added of industry j in region i in period t in the total value added of the industry, and F_ijt_ denotes the share of value added of industry j in region i in period t in the number of people employed in the industry.

#### 3.2.2. Explanatory variables: Human capital (HR) and technological progress (TP).

Human capital is a comprehensive indicator of the quantity, quality and structure of workers in an economic entity over a certain period of time.

Firstly, the human capital structure is characterised by the educational level of workers, and the proportion of human capital in each educational level to the overall human capital is taken as a component of the spatial vector, constituting a five-dimensional vector, and the angle between each vector and the base vector is calculated, and the angle of the vector represents the proportion of the human capital structure, and the basic expression is as follows:


$$h=\sum_{n=1}^{5}W_{j}\alpha_{j}$$ (7)

$$\alpha_{j}=\arccos(\frac{\sum_{n=1}^{5}\left(x_{j,n}x_{0,n}\right)}{\sqrt{\sum_{n=1}^{5}x_{j,n}^{2}}*\sqrt{\sum_{n=1}^{5}x_{0,n}^{2}}})$$ (8)

W_j_ denotes the weight of the pinch angle α_j_, x_j,n_ denote the nth component of each basic unit vector set X_j_=(j = 1,...,5),x_0,n_ denotes the nth component of the vector X_0_,The 5-dimensional human capital space vector is: X_1_=(1,0,0,0,0), X_2_=(0,1,0,0,0), X_3_=(0,0,1,0,0), X_4_=(0,0,0,1,0), X_5_=(0,0,0,0,1).

Second, to construct a substitution function covering different skill levels of labour under the given technological conditions [27], skill levels can be categorized into n levels, with the basic labour at the lowest skill level serving as the unit of measurement. The relative wages of high-skilled labour compared to basic labour indicate the quality of human capital possessed by skilled workers, so as to characterize the relationship between the different skill levels of labour in the economic output, and the basic expression is:


$$H=\phi (h_{1},h_{2},...,h_{n})$$ (9)

h_i_ denotes the human capital of skilled labour at level i,h_i_ = l_i_q_i_, i = 1,2,...,n, l_i_ denotes the number of labourers in the corresponding class.q_i_ denotes the quality of labour force, n denotes the skill level hierarchy, and ɸ denotes the aggregate function of human capital of labour force of different skill levels, and based on the substitution relationship between labour force of different skills, the model for calculating the total human capital in economic output is:


$$HR={\left[h_{1}^{(\theta -1)/\theta }+Z{\left(h_{2},h_{3},...,h_{n}\right)}^{(\theta -1)/\theta }\right]}^{\theta /(\theta -1)}$$ (10)

The elasticity of substitution (θ) represents the ease with which base level labour force human capital (h_1_) can be substituted by skilled labour force human capital (Z). This concept is crucial in understanding the flexibility of production processes and the adaptability of human capital to changing economic demands.

Technological progress (TP) reflects the technological innovation capacity of economic agents in a certain period of time, usually using the amount of invention patents granted (IPR) as a proxy variable, since a single indicator fails to capture the full dynamics of industrial technological progress, technological patent outcomes hinge on R&D inputs, with the intensity of R&D indicating the level of commitment to technological innovation, using the proportion of industrial R&D inputs in the industry’s economic output as a The R&D intensity (R&D) reflects the degree of investment in technological innovation, and the proportion of industrial R&D investment funds to industrial economic output is used as the calculation index. Therefore, This study utilizes the product of invention patents granted (IPR) and R&D intensity (R&D) as key indicators for assessing technological advancement, aligning with the understanding that patents reflect innovation and are outputs of R&D, and the basic expression is:


TP=IPR×(R&D) (11)

3.2.3. Control variables: size of industrial output (GDP), industrial expenditure (R&D), foreign direct investment (FDI).

### 3.3. Data sources and descriptive statistics

#### 3.3.1. Data sources.

This study selects data from the China Statistical Yearbook, China Agricultural Statistical Yearbook, China Industrial Statistical Yearbook, and China Tertiary Industry Statistical Yearbook spanning from 2008 to 2022, serving as the foundation for measuring various indicators. Missing data for certain years have been rectified and compensated for using the index smoothing method.

#### 3.3.2. Descriptive statistics.

The descriptive statistics of the main research variables in this paper are shown in Table 1, where the data reported in the table are standardised dimensionless processed values.

**Table 1:** Descriptive statistics of variables.

variant	maximum value	minimum value	average value	standard deviation
IR	1.365	0.427	0.891	1.033
TP	1.738	0.214	1.056	0.976
HR	0.367	0.026	0.205	1.019
GDP	3.792	1.215	1.876	0.801
R&D	1.335	0.269	0.734	1.161
FDI	1.847	0.087	1.202	1.005

## 4. Empirical testing

### 4.1. Panel vector auto-regression test

#### 4.1.1. Unit root test.

The results of the ADF unit root test are shown in Table 2, unit roots exist in all three variables: InIR, InTP, InHR. In the first order difference, ∆lnIR passes the test at the 1% significance level, while ∆InTP and ∆lnHR pass at the 5% significance level, and in the second order difference all three variables reject the original hypothesis at 1% significance level and pass the smoothing test.

**Table 2:** ADF unit root test for variables.

variant	ADF test value	ADF threshold			Forms of testing (C, T, n)	Test Conclusion
		1%	5%	10%		
lnIR	−1.697*	−3.382	−3.136	−2.877	(C, T, 1)	smoothing
lnTP	−2.335	−3.167	−2.702	−2.643	(C, T, 2)	smoothing
lnHR	−1.738	−2.664	−2.572	−2.341	(C, T, 3)	smoothing
∆lnIR	−1.823***	−3.211	−3.036	−2.784	(C, T, 0)	steadiness
∆lnTP	−2.384**	−2.725	−2.449	−2.381	(C, 0, 0)	smoothing
∆lnHR	−1.903**	−2.856	−2.604	−2.522	(C, T, 0)	smoothing
∆_2_lnIR	−2.018***	−2.731	−2.328	−1.824	(0, 0, 0)	steadiness
∆_2_lnTP	−3.143***	−2.508	−2.457	−2.412	(0, 0, 0)	steadiness
∆_2_lnHR	−2.721***	−2.453	−2.582	−1.877	(0, 0, 0)	steadiness

Note: ∆, ∆_2_ denotes first-order and second-order differences, respectively, ***, **, and * denote the rejection of the original hypothesis that the series is not stationary at the 1 percent, 5 percent, and 10 percent significance levels, respectively.

#### 4.1.2. Cointegration test.

The results of the traces test, as depicted in Table 3, indicate a statistically significant cointegration relationship between the variables at the 5% level. This outcome led to the rejection of the null hypotheses of no cointegration and at most one cointegration relationship. Conversely, the hypotheses suggesting at most two and three cointegration relationships were not rejected. These findings suggest a long-term and stable cointegration relationship among the variables lnIR, lnTP, and lnHR. The expression of these test results is as follows:

**Table 3:** Johansen cointegration test.

original hypothesis	eigenvalue (math.)	trace statistic	5%threshold value	p-value
None*	0.694	77.082	53.106	0.000
At most1*	0.208	25.736	16.817	0.003
At most2	0.024	12.452	6.021	0.089
At most3	0.016	4.497	3.828	0.702

Note: * indicates that the original hypothesis is rejected under 5% significance, None indicates that there is no cointegration in the model, and At most1 indicates that there is at most one cointegration in the model.


lnIR=13.538lnTP+3.672lnHR (12)

Based on the regression coefficient of formula (12), it can be determined that technological progress and human capital have a positive impact on industrial structural adjustment, and a 1% increase in technological progress affects industrial structural adjustment to the extent of 13.538%, while a 1% increase in human capital affects industrial structural adjustment to the extent of 3.672%, and technological progress has a greater impact on industrial structural adjustment, comparatively speaking, than human capital.

#### 4.1.3. Granger causality test.

The results of Granger test are shown in Table 4: (1) technological progress and industrial restructuring are causal, indicating that the innovation effect of technological progress can promote industrial restructuring, and industrial restructuring can force industrial technological innovation, which produces benign interaction between the two. (2) Technological progress is the cause of human capital, but human capital is not the cause of technological progress, indicating that technological progress can improve the quality of human capital and create favourable conditions for the effect of human capital ‘learning by doing’, however, human capital does not directly facilitate technological progress. (3) Industrial structural adjustment is not the precursor of human capital; rather, human capital serves as the impetus for industrial structural adjustment. This signifies that industrial structural adjustment can lead to concurrent shifts in the structure of human capital, and the mobility of industrial human capital can predict the direction of industrial structural adjustment.

**Table 4:** Granger causality test.

original hypothesis	F-statistic	p-value	conclude
Reasons why lnIR is not the Granger for lnTP	18.226	0.001	rejection
Reasons why lnTP is not the Granger of lnIR	11.713	0.003	rejection
Reasons why lnTP is not the Granger of lnHR	15.068	0.027	rejection
Reasons why lnHR is not the Granger of lnTP	8.349	0.024	non-rejection
Reasons why lnIR is not the Granger of lnHR	2.156	0.378	non-rejection
Reasons why lnHR is not the Granger of lnIR	9.744	0.016	rejection

#### 4.1.4. Estimation of regression results.

The cointegration test of human capital, technological progress and industrial restructuring found that the three variables were smooth on the second-order difference series, and the optimal lag order for constructing the PVAR model was determined to be 2 in Table 5 using the three determination criteria of Akaike Informativeness (AIC), Bayesian Informativeness (BIC), and Hannan-Quinn Informativeness (HQIC).

**Table 5:** PVAR model lag order test.

AIC	BIC	HQIC	hysteresis order (t)
−3.356	−2.628	−6.093	0
−6.762	−3.508	−9.114	1
−7.441*	−4.029*	−12.703*	2
−4.524	−3.313	−8.615	3

Note: * indicates the optimal number of lags under the corresponding discriminant criterion.

The test results of the panel vector auto-regressive model are shown in Table 6: (1) Technological progress and human capital still have a positive impact on industrial restructuring in lag two, and the regression coefficient of technological progress (0.743) is larger than that of human capital (0.191), indicating that technological progress has a greater impact on industrial restructuring, and technological progress (0.224) and human capital (0.107) in lag two also have a positive impact on the current period’s industrial restructuring, but the regression coefficient value is relatively small. The above results further verify that the innovation effect of technological progress and the accumulation effect of human capital are conducive to the optimisation and upgrading of industrial structure. (2) Human capital (−0.268) plays a substitution role for technological progress in the lagged period, and technological progress (−0.731) plays a substitution role for human capital in the lagged period, it is evident that there exists a certain degree of complementary between technical progress and human capital. Furthermore, technical progress can catalyze changes in the structure of human capital, while the ‘learning by doing’ effect and the knowledge spillover effect of human capital can enhance technical progress. Knowledge spillover effect can improve the efficiency of technical progress. According to the regression coefficient of the model, the expression of the equation of lag two is established as follows:

**Table 6:** Parameter estimates of panel vector auto-regressive models.

variant	lnIR	lnTP	_lnHR_
lnIR_(t-2)_	0.381 (2.039)	0.743 (1.358)	0.191 (0.214)
lnTP_(t-2)_	0.224 (1.193)	0.475 (2.408)	−0.268 (−1.772)
lnHR_(t-2)_	0.107 (2.843)	−0.731 (−2.469)	0.403 (2.581)
C	−4.224 (−2.301)	−2.573 (−1.427)	−2.606 (−2.388)
R^2^	0.725	0.733	0.701
F-statistics value	362.189	214.807	286.474


$$\left[\begin{array}{c} \ln IR_{t}\\ \ln TP_{t}\\ \ln HR_{t} \end{array}\right]=\left[\begin{array}{c} -3.784\\ -2.492\\ -2.511 \end{array}\right]+\left[\begin{array}{ccc} {}*20c0.381 & 0.743 & 0.191\\ 0.224 & 0.475 & -0.268\\ 0.107 & -0.731 & 0.403 \end{array}\right]*\left[\begin{array}{c} \ln IR_{\left(t-2\right)}\\ \ln TP_{\left(t-2\right)}\\ \ln HR_{\left(t-2\right)} \end{array}\right]+\left[\begin{array}{c} \omega_{1}\\ \omega_{2}\\ \omega_{3} \end{array}\right]$$ (13)

#### 4.1.5. Robustness test.

In order to improve the completeness of the panel vector auto-regressive model test and enhance the strength of the model interpretation, this study adopts the higher-order lag period test for effective determination, and introduces the lagged difference term in the regression model, the specific expression is:


$$\Delta y_{t}=\alpha +\beta t+\gamma y_{t-1}+\sum {\mathrm{\backslash nolimits}}_{i=1}^{p}\delta_{i}\Delta y_{t-1}+\varepsilon_{t}$$ (14)

**Fig 1:**
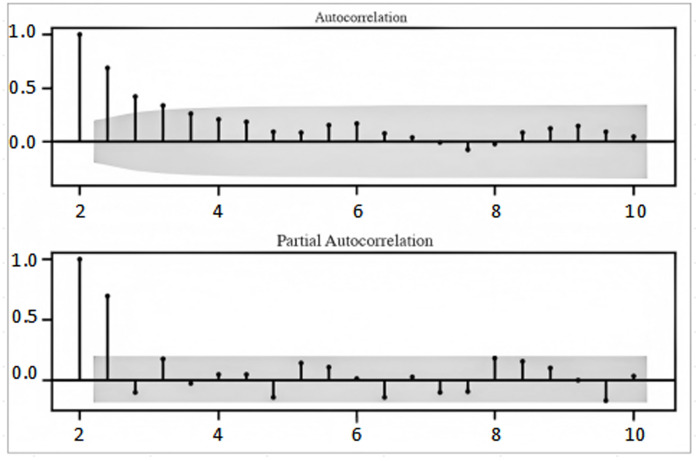
ACF plot of auto-correlation function.

Fig 1 shows that the model difference series data appears smooth, indicating that the higher order auto-correlation problem has been addressed.

The regression test results are shown in Table 7, the test values from ADF, LM, and FPE (Final Prediction Error) indicate that the P-value fails to meet the significance test criteria, the stability of the lagged order test model configuration has been verified.

**Table 7:** Higher order lag test.

hysteresis order (t)	ADF	p-value	LM	p-value	FPE
2	−3.518	0.274	4.326	0.346	7.36e-08
4	−3.773	0.505	6.174	0.423	7.84e-08
6	−3.785	0.538	6.608	0.471	5.06e-08
8	−4.249	0.611	8.787	0.529	4.67e-08
10	−5.018	0.842	11.436	0.644	4.33e-08

#### 4.1.6. Impulse response analysis.

**Fig 2:**
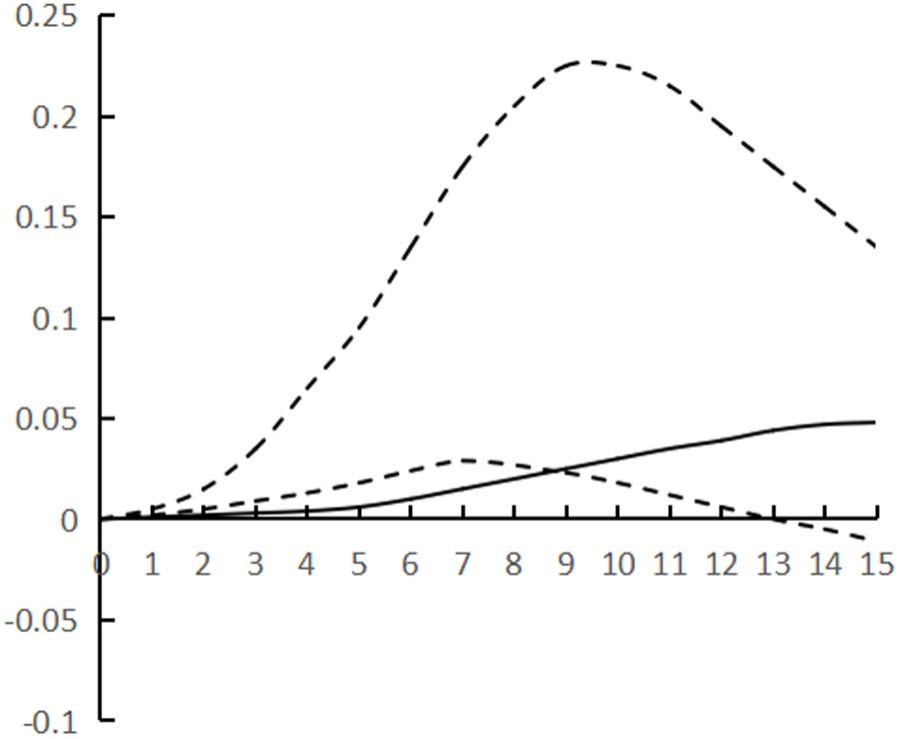
Impulse response of IR to TP.

**Fig 3:**
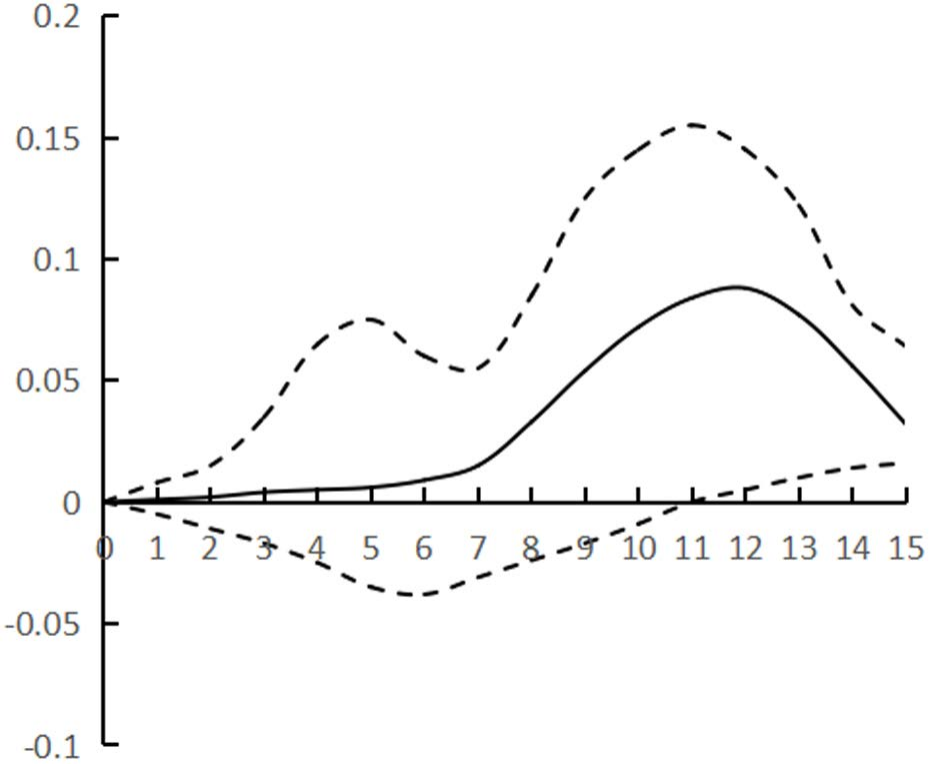
Impulse response of IR to HR.

**Fig 4:**
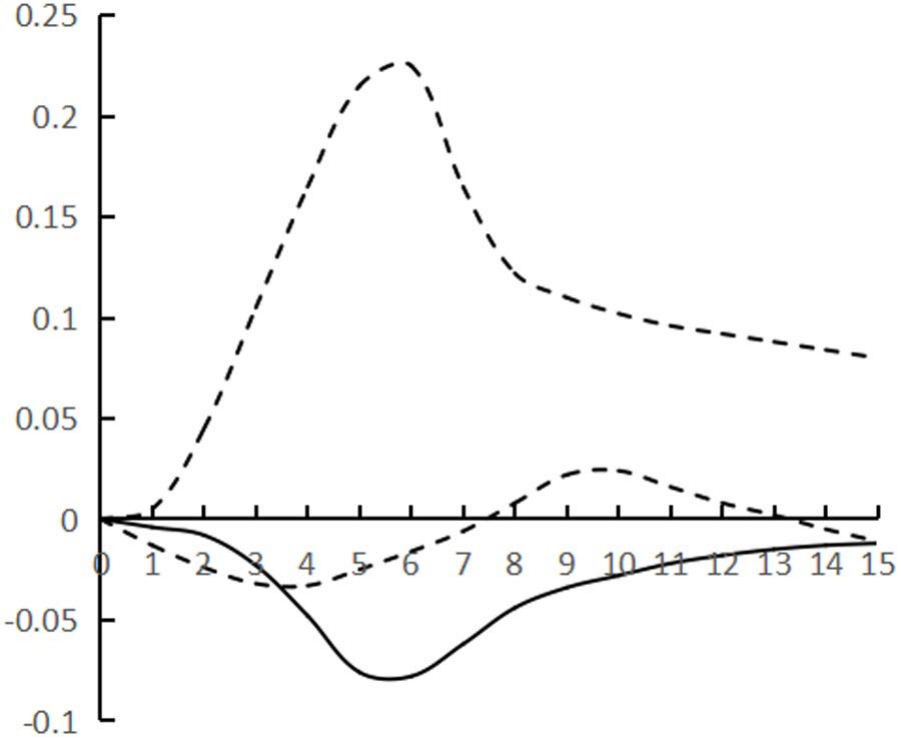
Impulse response of TP to IR.

**Fig 5:**
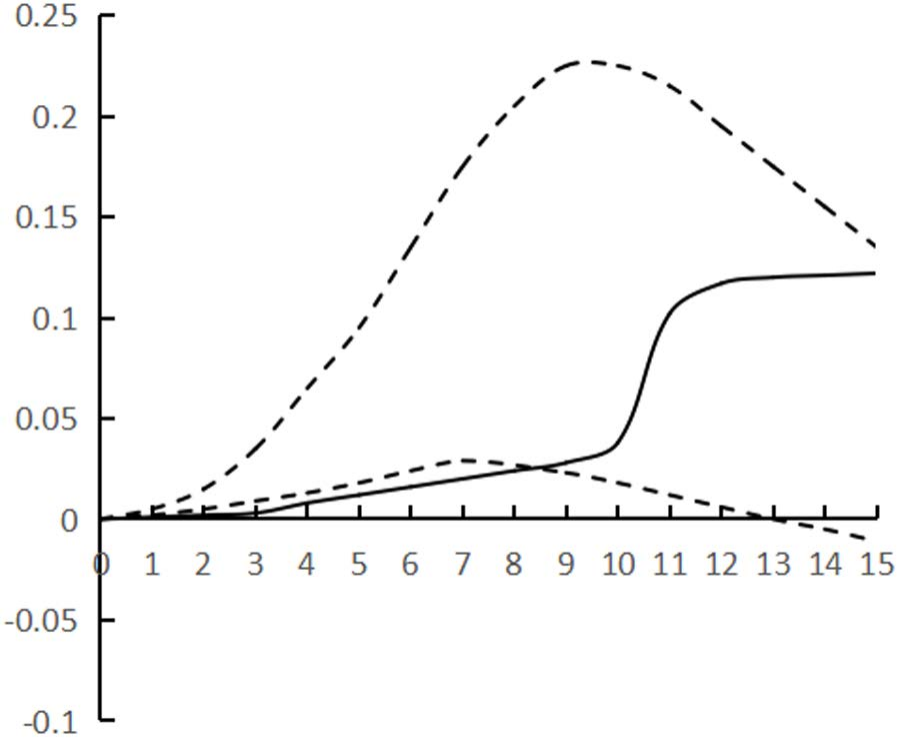
Impulse response of HR to IR.

**Fig 6:**
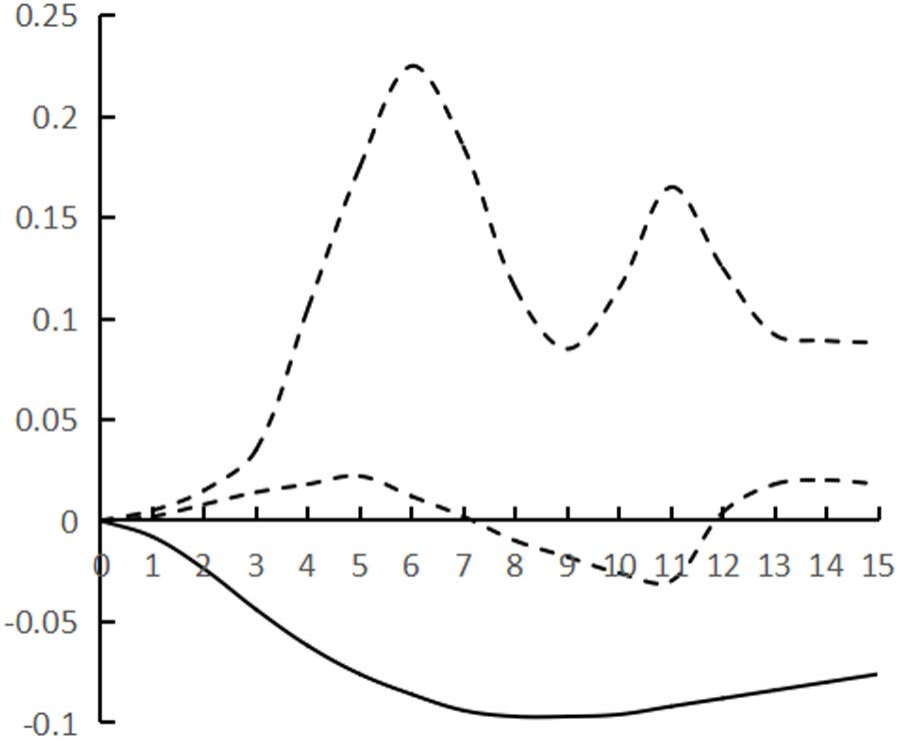
Impulse response of TP to HR.

**Fig 7:**
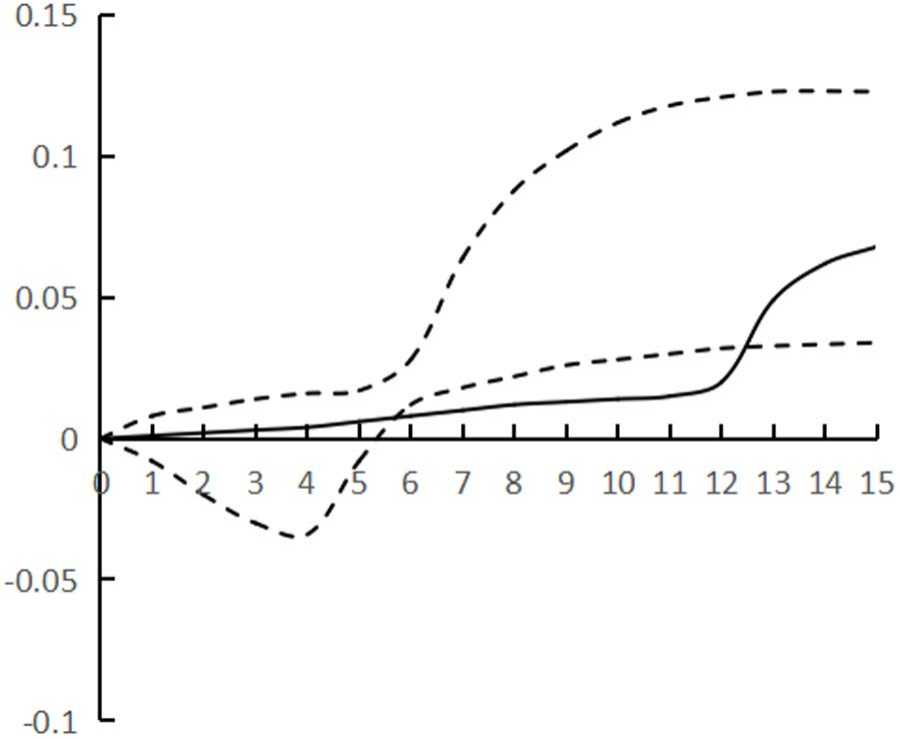
Impulse response of HR to TP.

Figs 2–7 plots the impulse response time dynamics of the three variables in the study period. Fig 2 demonstrates how industrial structural adjustment responds to technological progress. From period 1 to period 15, the impact of the former on the latter consistently displays a gradual upward trend, as the industrial structure undergoes rationalization and advancement, leading to a synchronous upgrade in the level of industrial technology.

Fig 3 shows the response of industrial structural adjustment to human capital, from the 1st to the 7th period, the impact of industrial structural adjustment on human capital is relatively flat, from the 8th to the 12th period, it shows a rapid rising trend and reaches the peak (0.088), and tends to decline after the 13th period, generally showing the characteristic of ‘slow and then fast and then fall back’.

Fig 4 shows the response of technological progress to industrial restructuring. From the first to the sixth period, the impact of technological progress on industrial restructuring shows a steady downward trend and reaches the trough (−0.078), and from the seventh to the fifteenth period it gradually and continuously rises, showing the characteristics of a ‘V’ shape.

Fig 5 shows the response of human capital to industrial structural adjustment, the first 10 periods basically maintain a slow upward trend, and the impact value is stable in the interval of [0–0.04], and from the 11th period onwards, it shows a ‘steep’ rise until the 15th period, peaking at 0.12, while the impact of human capital on industrial structural adjustment exhibits a pattern of ‘slow, then fast, and finally smooth’, indicating a ‘lag’ in human capital accumulation from quantitative to qualitative change, as well as a ‘lag’ in its impact on industrial structural adjustment. The influence of human capital on industrial structure adjustment shows the characteristic of ‘slow and then fast and then stable’, and the influence of human capital accumulation on industrial structure adjustment from variable to qualitative change has a ‘lag’.

Fig 6 shows the response of technological progress to human capital, and the impact of technological progress gradually strengthens from the 1st to the 8th period, and reaches the trough (−0.097) in the 9th period, and then rebounds steadily, presenting the characteristics of ‘U’, and the marginal substitution utility of technological progress to human capital ‘increases first and then decreases’, indicating that technological progress does not exhibit a consistent pattern of increasing and then decreasing, and its impact on industrial structure adjustment varies. The marginal substitution effect of technological progress for human capital initially increases and then decreases, indicating that technological progress cannot completely replace human capital, and after reaching the established threshold, the phenomenon of technological feedback to human capital may occur.

Fig 7 shows the response of human capital to technological progress, from the 1st to the 12th period of the degree of influence is relatively weak, the impact value remains in the [0–0.02] range, the latter 3 period of the impact value continues to rise and reach the peak (0.068), showing a ‘slow and then fast’ characteristics, the contribution of human capital to technological progress there is a ‘delayed’. The contribution of human capital to technological progress is ‘delayed’.

### 4.2. Hansen threshold test

Utilizing Hansen’s threshold regression model, this study examines human capital as the independent variable and technological progress as the threshold variable to assess their impact on industrial restructuring. The analysis delves into the mechanisms through which these factors influence industrial restructuring, which is further broken down into industrial structure rationalization (IR_m_) and industrial structure advancement (IR_q_). Empirical evidence from studies such as those referenced suggests that human capital plays a significant role in promoting industrial structure upgrading, particularly through channels like labor productivity and consumption level.

#### 4.2.1. Threshold regression based on rationalisation of industrial structure.

Bootsrap method is used to determine the number of threshold values, Table 8 gives the results of the threshold test based on rationalisation of industrial structure, the single threshold model passes the test at 10% significance level, the double threshold model passes the test at 1% significance level, and the triple threshold model does not pass the significance level test.

**Table 8:** Bootsrap test statistics.

threshold model	Number of draws	F-value	P-value	1%	5%	10%
single threshold	500	81.334*	0.022	19.085	16.767	11.944
double threshold	500	150.715***	0.000	28.624	21.309	14.738
tripartite threshold	500	103.457	0.218	12.198	9.484	7.371

Note: ***, **, * indicate that the test was passed at the 1 percent, 5 percent and 10 percent significance levels, respectively.

The threshold estimates and their corresponding 95% confidence intervals are detailed in Table 9, with a single threshold value of 0.308. and a confidence interval value of [0.287, 0.315], and double threshold values of 0.274 and 0.331, corresponding to confidence intervals of [0.259, 0.282] and [0.324, 0.345], respectively.

**Table 9:** Threshold values and confidence intervals based on rationalisation of industrial structure.

threshold model	threshold estimate	95 percent confidence interval
single threshold	0.308	[0.287, 0.315]
double threshold	0.274, 0.331	[0.259, 0.282] [0.324, 0.345]

**Fig 8:**
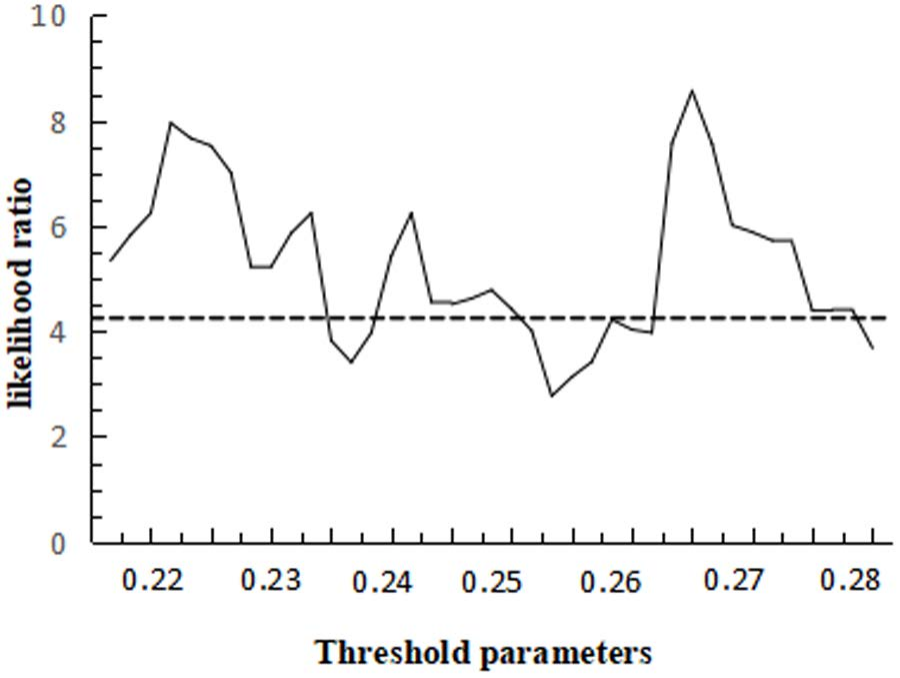
Plot of the likelihood ratio function for the first threshold.

**Fig 9:**
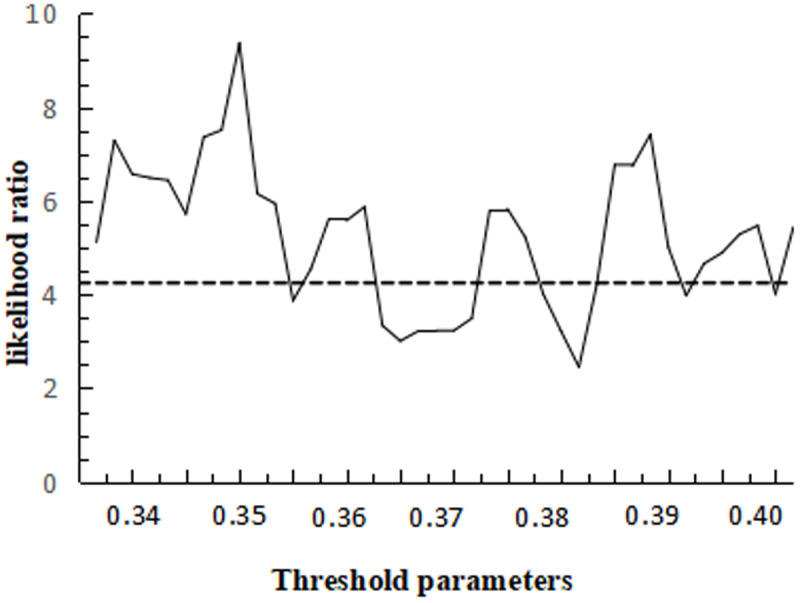
Plot of the second threshold likelihood ratio function.

Comparing the three threshold models, the double threshold test results are optimal, and the likelihood ratio function plots of the two threshold values under the double threshold model are given in Figs 8 and 9, with the LR critical value of 4.27.

The results in Table 10 show that technological progress has a significant role in promoting the rationalisation of the industrial structure, and that the positive impact of human capital on the rationalisation of the industrial structure is premised on technological progress. (1) In the single threshold model, when the threshold value of technological progress is lower than 0.308, the regression coefficient value of human capital is 0.114, and when the threshold value of technological progress is greater than 0.308, the regression coefficient value of human capital is 0.163. (2) In the double threshold model, as technological progress surpasses the thresholds of 0.274 and 0.331, the regression coefficient for human capital rises from 0.027 to 0.228, signifying that human capital continues to positively influence the rationalization of industrial structure. This effect is particularly pronounced when investments in technological research and development, as well as technological innovation capabilities, are substantial. The empirical study from Sichuan province highlights the significant impact of human capital, especially researchers, on the output of technological innovation, as evidenced by the high elasticity of patent applications and new product sales to the number of researchers. This complements the understanding that human capital investment and technological progress are mutually reinforcing, with human capital providing essential talent support for technological advancement and technological progress creating opportunities for human capital development. Moreover, human capital absorption is crucial for the adoption and transformation of technological progress, as emphasized in the analysis of developing countries. It is conducive to accelerating the process of industrial structure rationalisation.

**Table 10:** Threshold model regression estimates based on rationalisation of industrial structure.

single threshold			double threshold		
variant	regression coefficient	P-value	variant	regression coefficient	P-value
TP	0.371***	0.003	TP	0.405***	0.000
HR (TP ≤ 0.308)	0.114*	0.082	HR (TP ≤ 0.274)	0.027	0.528
HR (TP > 0.308)	0.163***	0.001	HR (0.274 < TP ≤ 0.331)	0.043*	0.054
			HR (TP > 0.331)	0.228***	0.000

Note: ***, **, * indicate that the test was passed at the 1 percent, 5 percent and 10 percent significance levels, respectively.

#### 4.2.2. Threshold regression based on advanced industrial structure.

Threshold regression test steps for industrial structure advancement are the same as above, Bootsrap method was again used to determine the number of threshold values and the results of the threshold test based on the advanced industrial structure are given in Table 11, where the single threshold model passes the test at 1% significance level and the double threshold model fails the significance level test.

**Table 11:** Bootsrap test statistics.

threshold model	Number of draws	F-value	P-value	1%	5%	10%
single threshold	500	90.257***	0.000	28.914	18.602	10.116
double threshold	500	73.018	0.351	20.532	11.484	8.607

Note: ***, **, * indicate that the test was passed at the 1 percent, 5 percent and 10 percent significance levels, respectively.

The results in Table 12 show that: there is a single threshold effect of human capital on industrial structure advancement, the estimated value of threshold is 0.382, the 95% confidence interval is [0.367, 0.388].

**Table 12:** Threshold values and confidence intervals based on advanced industrial structure.

threshold model	threshold estimate	95 percent confidence interval
single threshold	0.382	[0.367, 0.388]

**Fig 10:**
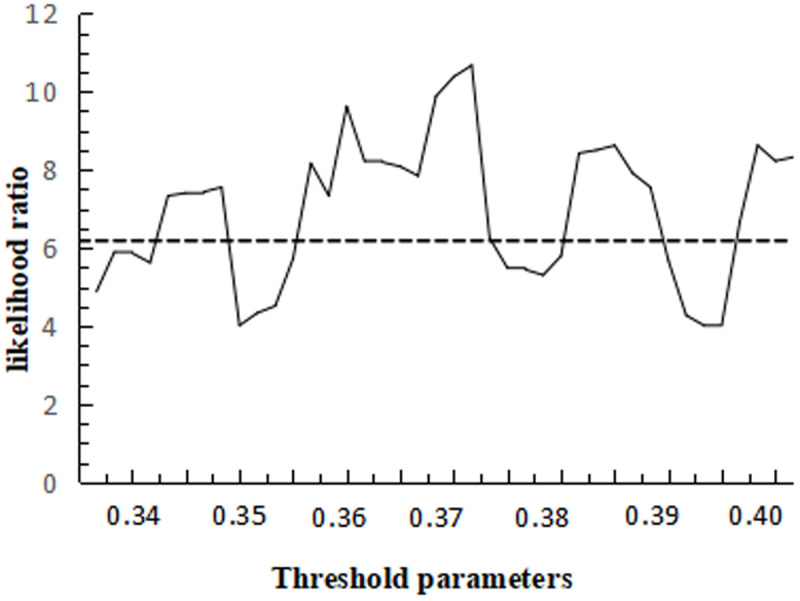
Plot of the likelihood ratio function for the threshold value of the single threshold model.

Fig 10 gives a plot of the likelihood ratio function of the threshold values under the single threshold model,and the critical value of LR is 6.12.

The findings in Table 13 indicate that technological advancement significantly contributes to the progression of industrial structure, as evidenced by the regression coefficients.When the threshold value of technological advancement is below 0.382, human capital negatively influences the advancement of industrial structure. However, once the threshold is surpassed, human capital begins to positively affect industrial structure advancement. value of 1.803, when the threshold value of technological progress is lower than 0.382, human capital has a negative impact on the industrial structure advanced, after crossing the threshold value, human capital has a positive impact on the industrial structure advanced, and the value of the regression coefficient changes from −0.235 to 0.771, indicating that from the rationalization of the industrial structure to its advanced state, the level of industrial technological progress remains a limiting factor, and it is important to To exert the positive influence of human capital on industrial structure upgrading, there is still a technological threshold, therefore, it is imperative to propel technological advancements through the upgrading of industrial structure, subsequently enhancing human capital quality to achieve an optimal synergy between the two, and the results of this study strongly confirm the theoretical viewpoint that economically late-developed countries and regions give priority to the theory of ‘industrial structure upgrading’.

**Table 13:** Threshold model regression estimates based on advanced industrial structure.

single threshold		
variant	regression coefficient	P-value
IT	1.452***	0.000
HR (IT ≤ 0.374)	−0.318*	0.024
HR (IT > 0.374)	0.743***	0.003

Note: ***, **, * indicate that the test was passed at the 1 percent, 5 percent and 10 percent significance levels, respectively.

## 5. Research findings and policy implications

### 5.1. Findings

Based on theoretical explanations and empirical tests concerning human capital, technological progress, and industrial restructuring, the following research conclusions are drawn:

Firstly, technological progress and industrial restructuring are causal. The impulse response relationship of the three variables in the research cycle: the impact of human capital on industrial structural adjustment shows ‘slow and then fast and then smooth’, the impact of technological progress on industrial structural adjustment shows ‘V’ characteristics, and the impact of technological progress on human capital shows ‘U’ characteristics.

Secondly, both human capital and technological progress facilitate industrial structure adjustment, with human capital influencing this adjustment through technological progress serving as a threshold variable, which is specifically manifested in The dual threshold effect of technological progress influences the impact of human capital on the rationalization of industrial structure, while the single threshold effect of technological progress affects the influence of human capital, on the influence of industrial structure sophistication.

Thirdly, as the industrial structure adjustment transitions from rationalization to advancement, the threshold for technological progress continues to rise, emphasizing the capacity of human capital to absorb and transform technological innovations. By fully leveraging the synergistic effect between human capital and technological innovation, and enhancing their compatibility and adaptability, we can accelerate the upgrading of the industrial structure.

### 5.2. Policy implications

Firstly, differentiate the path of industrial structure upgrading according to local conditions. China’s regional economic development exhibits imbalances, with varying directions in industrial structure adjustment evident. The eastern region should focus on the spatial layout of high-precision industry chain, lead the high-end positioning of industry with the advantage of technological innovation agglomeration, and improve the quality of advanced industrial structure. The central and western regions should base on the characteristics of local leading industries, drive the synergistic development of related industries with the advantages of regional resource endowment, and accelerate the process of rationalisation of industrial structure.

Secondly, we should grasp the triggering mechanism of technological innovation precisely according to the time. China’s industrial restructuring should seamlessly integrate big data, artificial intelligence, cloud computing, and other core technologies, fostering a robust integration effect in industrial technological innovation, shorten the cycle of technological research and development, application and promotion, and form a forcing mechanism for technological progress to lead the industrial revolution, so as to Leverage the continuous iteration of technology to enhance the total factor productivity (TFP) of the industry, as evidenced by the growth in labor productivity and the increase in capacity utilization rates, and realise the new pattern of new technology reforming traditional industries, consolidating the dominant industries, and generating the new industries.

Thirdly, according to the conditions of production, scientifically tapping the potential of human capital dividend. China has transitioned from relying on population dividend to harnessing human capital dividend, seamlessly integrating basic education, vocational training, higher education, and continuing learning, with a strong emphasis on enhancing human capital quality. Establish a dynamic matching mechanism between the demand side of industrial talents and the supply side of the human capital market to improve the efficiency of human capital allocation. Leverage the combined strengths of an effective market and an active government to effectively steer the allocation of industrial human capital.

Fourth, synergise and optimise, build a matching mechanism between human capital, technological progress and industrial restructuring. For countries or regions undergoing economic restructuring, establishing the concept of industrial upgrading is paramount. Firstly, the profound transformation of industrial structure should drive the evolution of technological innovation and the realignment of human capital structure. Secondly, we should strengthen investment in national education, consolidate the material foundation of human capital, and give full play to the ‘learning by doing’ effect of industrial human capital.Lastly, we should take the initiative to adapt to the new scientific and technological revolution, boost industrial technological innovation capabilities and nurture human capital through technological advancements, direct human capital to surpass technological barriers, and accelerate the rationalization and upgrading of the industrial structure.

### 5.3. Research shortcomings and prospects

Based on the theoretical literature and academic perspective, this study utilizes Chinese industry-level data to investigate the causal relationship and threshold effect among human capital, technological progress, and industrial structure adjustment. This contributes to enriching the theoretical research on industrial structure optimization and upgrading from the perspective of production factors. However, due to model design limitations, further exploration is not conducted.he mechanism of the role of the three, and in regard to the threshold effect of technological progress, the research variables may also have interaction effects and moderating and mediating effects, which can provide the corresponding ideas for subsequent in-depth research. For the threshold effect of technological progress, the research variables may also have interaction effects, moderating and mediating effects, etc., which also provides corresponding ideas and theoretical basis for subsequent in-depth research.

## Supporting information

S1 File Research data. (XLS)
